# Tau and apolipoprotein E modulate cerebrovascular tight junction integrity independent of cerebral amyloid angiopathy in Alzheimer’s disease

**DOI:** 10.1002/alz.12104

**Published:** 2020-08-22

**Authors:** Chia-Chen Liu, Yu Yamazaki, Michael G. Heckman, Yuka A. Martens, Lin Jia, Akari Yamazaki, Nancy N. Diehl, Jing Zhao, Na Zhao, Michael DeTure, Mary D. Davis, Lindsey M. Felton, Wenhui Qiao, Yonghe Li, Hongmei Li, Yuan Fu, Na Wang, Melissa Wren, Tomonori Aikawa, Marie-Louise Holm, Hiroshi Oue, Cynthia Linares, Mariet Allen, Minerva M. Carrasquillo, Melissa E. Murray, Ronald C. Petersen, Nilüfer Ertekin-Taner, Dennis W. Dickson, Takahisa Kanekiyo, Guojun Bu

**Affiliations:** 1 Department of Neuroscience, Mayo Clinic, Jacksonville, Florida, USA; 2 Division of Biomedical Statistics and Informatics, Mayo Clinic, Jacksonville, Florida, USA; 3 Department of Neurology, Mayo Clinic, Rochester, Minnesota, USA; 4 Department of Neurology, Mayo Clinic, Jacksonville, Florida, USA

**Keywords:** Alzheimer’s disease, blood-brain barrier, cerebral amyloid angiopathy, tau, tight junction

## Abstract

**Introduction:**

Cerebrovascular pathologies including cerebral amyloid angiopathy (CAA) and blood-brain barrier (BBB) dysregulation are prominent features in the majority of Alzheimer’s disease (AD) cases.

**Methods:**

We performed neuropathologic and biochemical studies on a large, neuropathologically confirmed human AD cohort (N = 469). Amounts of endothelial tight junction proteins claudin-5 (CLDN5) and occludin (OCLN), and major AD-related molecules (amyloid beta [A*β*40], A*β*42, tau, p-tau, and apolipoprotein E) in the temporal cortex were assessed by ELISA.

**Results:**

Higher levels of soluble tau, insoluble p-tau, and apolipoprotein E (apoE) were independently correlated with lower levels of endothelial tight junction proteins CLDN5 and OCLN in AD brains. Although high A*β*40 levels, *APOE ε*4, and male sex were predominantly associated with exacerbated CAA severity, those factors did not influence tight junction protein levels.

**Discussion:**

Refining the molecular mechanisms connecting tau, A*β*, and apoE with cerebrovascular pathologies is critical for greater understanding of AD pathogenesis and establishing effective therapeutic interventions for the disease.

## 1 | INTRODUCTION

Alzheimer’s disease (AD) is the most common form of dementia in the elderly accounting for between 60% and 80% of all cases.^1^ While AD is characterized by neuropathologies related to brain accumulation of amyloid beta (A*β*) and tau as well as progressive neurodegeneration,^2^ cerebrovascular lesions including cerebral small vessel disease (cSVD) and cerebral amyloid angiopathy (CAA) are also detected in the majority of AD cases.^3,4^ Accumulating evidence has shown that risk factors for the vascular cognitive impairment and dementia (VCID) spectrum such as hypertension, diabetes mellitus, atrial fibrillation, hypercholes-terolemia, and reduced physical activity are associated with increased risk for AD.^5,6^ Indeed, VCID often co-exists with AD in which cases presenting with mixed pathologies of both are estimated to represent 50% to 70% of all dementia.^6,7^ Furthermore, the effect of cerebrovascular and amyloid pathologies on cognition is likely additive rather than synergistic.^8^ Thus, determining the cerebrovascular contribution to AD is of growing importance in exploring the pathogenic heterogeneity in AD dementia.

The major cause of VCID is cSVD affecting the cerebral arterioles, capillaries, and venules,^9^ in which endothelial cell dysregulation likely triggers the pathogenic cascade. Endothelial damage leads to increased cerebrovascular permeability and neuroinflammation resulting in thickening and stiffness of the vessel wall, which eventually causes impaired autoregulation as well as luminal narrowing and occlusion.^10^ In brain capillaries, endothelial cells uniquely construct tubular structures forming the blood-brain barrier (BBB), surrounded by vascular mural pericytes and astrocytic end-feet.^11–13^ To maintain a sealed environment for the brain, the BBB relies on tight junctions which comprise a number of proteins; among them, claudin-5 (CLDN5) and occludin (OCLN) have been shown to be the key transmembrane proteins that regulate endothelial barrier integrity.^14^ BBB leakage is an early neuropathologic event in the development of white matter hyper-intensities (WMHs), one of the main magnetic resonance imaging (MRI) features of cSVD.^15,16^ Of note, BBB dysfunction has also been shown to contribute to the pathogenesis of AD.^17,18^ Our previous work has demonstrated that endothelial tight junction proteins, which are key components in maintaining BBB integrity, were specifically reduced in the cortical regions during AD progression.^19^ Additionally, CAA is a representative cerebrovascular pathology observed in most AD cases.^20,21^ In CAA, A*β*40 predominantly deposits in the smooth muscle cell layers of leptomeningeal/cortical arteries and capillaries,^22–25^ which is exacerbated in males and in apolipoprotein E (*APOE*) *ε*4 carriers in AD.^21^ CAA is responsible for ~20% of cerebral hemorrhage cases in the elderly and increases the risk of hemorrhage as assessed by MRI.^20,26^ Although BBB damage and CAA are main cerebrovascular phenotypic features frequently observed in AD, their relationship is not precisely understood.

Interestingly, recent neuroimaging studies have revealed that vascular risk factors or cSVD associate with increased brain accumulation of both tau and A*β* in cognitively unimpaired older individuals^27^ and VCID patients.^28^ Moreover, apoE isoforms are also critically involved in A*β* and tau pathologies.^29–32^ Therefore, in this study, we biochemically assessed how the amounts of two major tight junction proteins, CLDN5 and OCLN, are correlated with CAA severity and the levels of several AD-related molecules, including A*β*, tau, p-tau, and apoE in the temporal cortex of a large neuropathologically confirmed AD cohort.

## 2 | MATERIALS AND METHODS

### 2.1 | Human autopsied AD brain samples

Neuropathologically confirmed *post mortem* AD brain tissue was obtained from the Mayo Clinic Brain Bank for neurodegenerative diseases. A total of 469 AD cases were identified for inclusion in this study. All cases in this cohort are non-Hispanic white decedents. Cases with comorbid neuropathologies were not excluded to represent the broad spectrum of an autopsied AD population. Experimental procedures were conducted in accordance with protocol approved by the Mayo Clinic Institutional Review Board. All cases underwent standardized neuropathologic sampling and evaluation as previously described.^33^ Thioflavin-S fluorescent microscopy was used to evaluate AD neuropathologic change, including Braak tangle stage and Thal amyloid phase, as previously described in detail.^34–36^ Thioflavin-S microscopy was also used to assess CAA severity and was scored in the superior temporal cortex, inferior parietal cortex, middle frontal cortex, motor cortex, and visual cortex using a semi-quantitative method as follows: 0, no amyloid positive vessels; 0.5, Amyloid deposition restricted to the leptomeninges; 1, mild amyloid deposition observed in both leptomeninges and parenchymal vessels; 2, moderate circumferential amyloid deposition in some vessels; 3, widespread severe amyloid deposition in leptomeninges and parenchymal vessels; 4, even more severe CAA with dyshoric changes noted.^19^ The averaged CAA scores were quantified from these five cortical regions. Age at death, sex, *APOE ε*4 status, Braak stage (neurofibrillary tangle), Thal phase (amyloid load), and CAA score is summarized in Table 1. Genomic DNA was extracted from frozen brain tissue using the standard protocols. Geno-typing for *APOE* single nucleotide variants (rs429358 C/T and rs7412 C/T), which define the *APOE ε*2, *ε*3, and *ε*4 alleles, was performed using custom TaqMan Allelic Discrimination Assays on a QuantStudio 7 Flex Real-Time PCR system (Applied Bio-Systems, Foster City, CA, USA). Genotype calls were made using TaqMan Genotyper Software v1.3 (Applied Bio-Systems). Genotype call rates were 100% for each variant and there were no departures from Hardy-Weinberg equilibrium (all *P* > .01). The distribution of *APOE* genotypes was as follows: *ε*2/*ε*3, n = 6 (1.3%); *ε*2/*ε*4, n = 14 (3.0%); *ε*3/*ε*3, n = 152 (32.4%); *ε*3/*ε*4, n = 230 (49.0%); and *ε*4/*ε*4, n = 67 (14.3%).

### 2.2 | Sample preparation

Superior temporal cortex tissues of 469 cases dissected from AD brains were subjected to three-step sequential extractions in Tris-buffered saline (TBS) buffer, detergent-containing buffer (TBS/1% Triton X-100, termed TX), and formic acid (FA) as previously described with minor modification.^21^ After removal of meninges and blood vessels, 100 to 120 mg of frozen brain tissue were homogenized in ice-cold TBS buffer containing a protease inhibitor cocktail (Roche) and a phosphatase inhibitor (Roche) by Polytron homogenizer. After centrifugation at 100,000 × g for 60 minutes at 4°C, the supernatant was collected (referred to as TBS fraction). The residual pellet was re-homogenized in TX buffer with protease and phosphatase inhibitors, sonicated, incubated at 4°C for 30 minutes with end-over-end agitation, and centrifuged as above. The resultant supernatant was referred to as TX fraction. The pellet was re-suspended in 70% formic acid, sonicated, and incubated for 12 to 16 hours at 4°C. After centrifugation as above, the resultant supernatant (referred to as the FA fraction) was collected and neutralized 20-fold with 1 M Tris-buffer (pH 11). All fractions were aliquoted and stored at −80°C until use.

### 2.3 | Quantification of tight junction proteins and AD-related proteins

The amount of CLDN5 was determined by enzyme-linked immunosorbent assay (ELISA) as described in our published work^19^ with minor modification. A mouse monoclonal CLDN5 (4C3C2) antibody (Invitrogen; 35–2500) was used as the capture antibody and a recombinant anti-CLDN5 antibody (Abcam; ab131259) was used as the detection antibody. Goat anti-rabbit IgG antibody (Invitrogen) was used as the secondary antibody for the CLDN5 ELISA. The amount of OCLN was examined by using a rabbit polyclonal antibody (Abcam, ab31721) as a capture antibody and a horseradish peroxidase (HRP)-conjugated mouse monoclonal antibody (Invitrogen, OC-3F10) as the detection antibody. Recombinant CLDN5 and OCLN proteins (Novus Biologicals) were used as standards. The amount of endothelial cell marker, CD31 was determined by a commercial ELISA kit (R&D) according to the manufacturer’s instruction with minor modifications. The membrane proteins were measured in the TX fraction and normalized by (ie, divided by) the levels of the endothelial cell marker CD31 to minimize the potential influence of different cerebrovascular density among samples. These CLDN5/CD31 and OCLN/CD31 measures are subsequently referred to as simply CLDN5 and OCLN, respectively.

Secreted proteins, membrane proteins, and the neuropathologic aggregated proteins for A*β*40, A*β*42, apoE, and tau were measured from TBX, TX, and FA fractions by ELISAs as previously described.^19,21,37^ In brief, A*β*40 and A*β*42 levels were measured using end-specific monoclonal antibodies (13.1.1 for A*β*40; 2.1.3 for A*β*42) and a HRP-conjugated detection antibody (Ab5; human A*β*1-16 specific). All A*β* antibodies were in-house-produced by Mayo Clinic. The levels of apoE were determined by ELISA using a goat anti-apoE antibody (AB947; Millipore) as a capture antibody and biotin-conjugated goat anti-apoE antibody (K74810B; Meridian Life Sciences) as a detecting antibody. Levels of tau were determined by ELISA using a monoclonal tau antibody (HT7; Thermo Scientific) as a capture antibody and a biotin-conjugated tau antibody (BT2; Thermo scientific) as a detection antibody. The data of spike-and-recovery and linearity-of-dilution assessment for ELISAs were shown in Table S1 in supporting information. Phospho-tau (Thr231) was determined by Phospho-Tau Kit (Meso Scale Discovery) according to the manufacturer’s instruction.

AD-related outcome measures including CAA score, A*β*40 (TBS, TX, and FA), A*β*42 (TBS, TX, and FA), apoE (TBS, TX, and FA), total tau (TBS, TX, and FA), and phospho-tau (p-tau; TBS, TX, and FA) were examined. All measured values by ELISA were normalized against total protein amounts. In addition, the intra- and inter-assay coefficients of variation on the measurements were summarized in Table S2 in supporting information.

### 2.4 | Statistical analysis

Unadjusted pair-wise correlations between variables were assessed using Spearman’s test of correlation; *P*-values <.05 were considered statistically significant in this exploratory analysis. Variables were transformed as needed in all subsequently described regression analysis due to skewed distributions (Table S3 in supporting information).

Associations of AD-related molecules with CLDN5, OCLN, and CAA score (and also associations of CLDN5 and OCLN with CAA score) were evaluated using single-variable and multivariable linear regression models. Multivariable models were adjusted for age at death, sex, CAA score, number of *APOE ε*4 alleles, Braak stage, and Thal phase. Regression coefficients (referred to as *β*) and 95% confidence intervals (CIs) were estimated. *P*-values <.0028 (associations with CLDN5 and OCLN) and <.0025 (associations with CAA score) were considered statistically significant after applying a Bonferroni correction for multiple testing separately for each outcome measure.

Associations of sex and presence of the *APOE ε*4 allele with CLDN5 and OCLN were evaluated using the previously described linear regression models, where *P*-values <.025 were considered statistically significant after Bonferroni correction for multiple testing separately for each tight junction protein measure. Additionally, given evidence of associations of total tau, p-tau, and apoE with CLDN5 and OCLN, we also performed several interaction analyses. First, we examined interactions of sex and presence of *APOE ε*4 with each of total tau, p-tau, and apoE regarding associations with CLDN5 and OCLN using multivariable linear regression models with the aforementioned model adjustments. After adjusting for multiple testing using a Bonferroni correction separately for each tight junction protein measure, *P*-values <.0056 were considered statistically significant. Second, we examined the interaction between total tau TBS and apoE TBS in multivariable linear regression analysis, adjusting for the aforementioned potential confounding variables, and where a *P*-value <.05 was considered significant.

We examined associations of sex and presence of *APOE ε*4 with CAA score using the previously described linear regression models; *P*-values <.025 were considered significant after using a Bonferroni adjustment for multiple testing. Associations of sex and *APOE ε*4 with A*β*40, A*β*42, A*β*40/A*β*42 ratio, apoE, total tau, and p-tau levels were also evaluated using multivariable linear regression models; *P*-values <.0083 were considered statistically significant after correcting for multiple testing. Statistical analyses were performed using SAS (version 9.4)

## 3 | RESULTS

### 3.1 | Tau and apoE are negatively associated with tight junction proteins in the temporal cortical region of AD brains

To gain insight into how the expression of tight junction proteins is altered in AD, we quantified the levels of AD-related molecules including A*β*, tau, p-tau, and apoE, and two major tight junction proteins, CLDN5 and OCLN, by ELISAs in a large sample cohort of autopsy-confirmed AD cases (N = 469; Table 1). Unadjusted pair-wise correlations between all measures (tight junction proteins and AD-related molecules) were first evaluated in an exploratory analysis (Table 2). Notably, the two tight junction proteins, CLDN5 and OCLN, were positively correlated with one another (Spearman’s *r*: 0.48, *P* < .001). Multivariable regression analysis revealed that lower levels of CLDN5 were associated with higher levels of A*β*42 (TBS), total tau (TBS and TX), p-tau (FA), and apoE (TBS, TX, and FA; all *P* < .001, Table 3), as well as with lower levels of p-tau (TX, *P* = .001, Table 3). For OCLN, a lower value was associated with higher levels of A*β*40/A*β*42 (TX), total tau (TBS), and apoE (TBS and TX), but with lower values of A*β*40 (FA), A*β*42 (TX and FA), and p-tau (TX) in multivariable analysis (all *P* ≤ .002, Table 3). The most consistent associations with tight junction proteins were observed for soluble tau (TBS) and apoE (TBS and TX), implying a neuropathologic role of tau and apoE in tight junction integritybrk in AD.

Neither CLDN5 nor OCLN was significantly associated with sex or *APOE ε*4 (all *P* ≥ .025, *P* < .025 considered significant after multiple testing adjustment). Given that associations with tight junction proteins were observed for total tau, p-tau, and apoE, we next examined how *APOE ε*4 status and sex influence these associations. No interactions of sex or *APOE ε*4 with tau, p-tau, or apoE were detected with respect to associations with tight junction proteins (all *P* ≥ .017, *P* < .0056 considered significant after multiple testing adjustment).

### 3.2 | Higher levels of soluble tau, insoluble p-tau, and apoE are independently associated with reduction of tight junction proteins in AD brains

**FIGURE 1:**
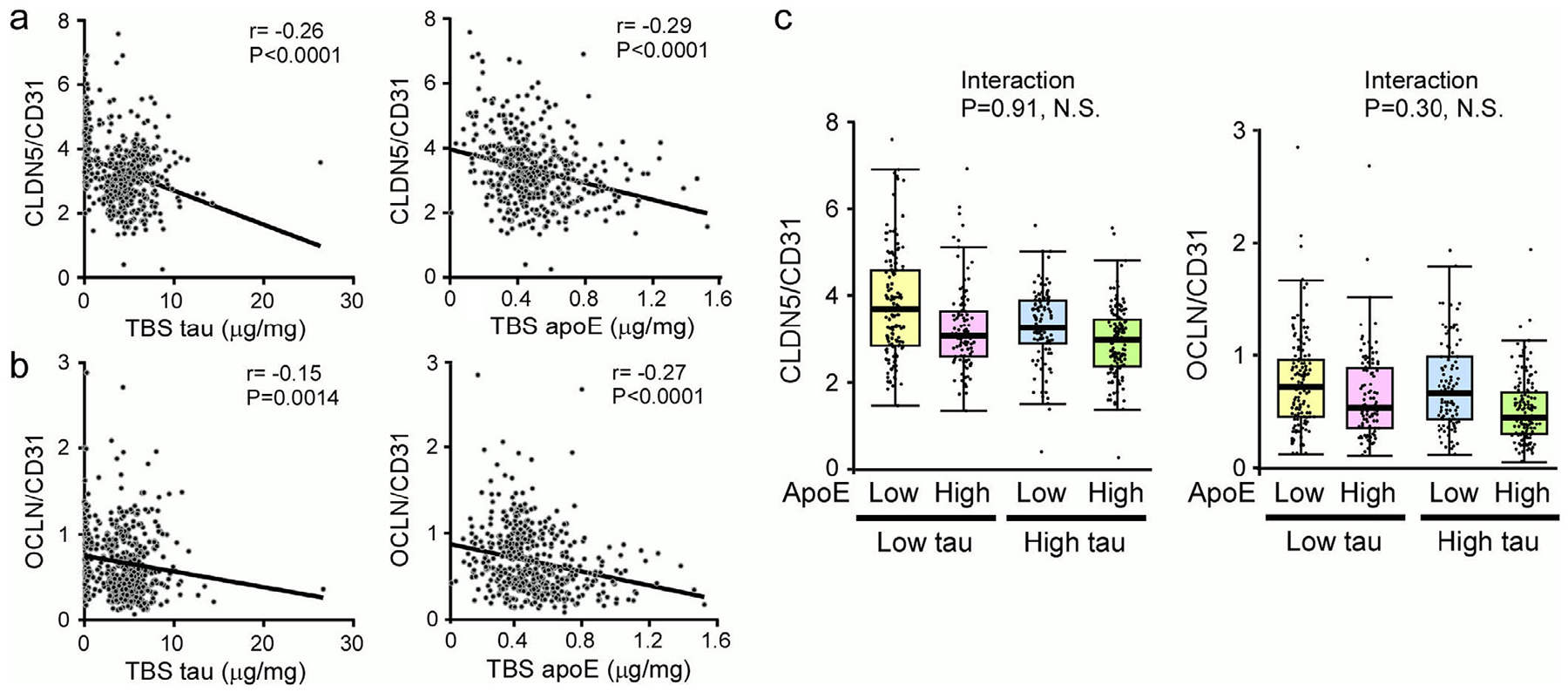
Negative association between tight junction proteins and soluble tau or apolipoprotein E (apoE). The claudin-5 (CLDN5)/CD31 (A) or occludin (OCLN)/CD31 (B) levels from temporal cortex of Alzheimer’s disease (AD) cases were plotted against soluble tau and soluble apoE levels. The correlation between tight junction proteins and tau or apoE (TBS fractions) was evaluated using Spearman’s correlation analysis. *P*-values <.0028 were considered statistically significant. An estimated regression line is shown on each figure to enhance visualization of negative correlations. C, Boxplots showing the levels of CLDN5/CD31 or OCLN/CD31 from AD cases according to soluble tau and apoE, both of which were dichotomized as low or high based on the sample median for ease of presentation. NS, not significant. Note the independent effects of soluble tau and apoE on tight junction protein levels

Greater levels of tau and apoE were associated with a lower CLDN5 and OCLN, particularly in the TBS fraction (Figure 1A and B). We therefore examined possible independent and interactive effects of soluble tau and apoE for association with tight junction proteins. Multivariable linear regression analysis adjusting for both tau and apoE (in addition to the previously mentioned potential confounding variables) identified association of both measures with CLDN5 (tau, *β*: −0.35, *P* < .001; apoE, *β*: −0.26, *P* < .001) and OCLN (tau, *β*: −0.03, P = 00046; apoE, *β*: −0.06, *P* < .001). This indicates that the effects of total tau and apoE on tight junction proteins are independent of one another. Subsequently, we examined whether tau and apoE may interact in association with tight junction proteins. We did not observe a statistically significant interaction between tau and apoE with respect to associations with CLDN5 (*P* = 0.91) or OCLN (*P* = .30). The consistent reductions in tight junction proteins in patients with higher tau level regardless of apoE level (and vice versa) are further illustrated in Figure 1C, where for ease of presentation both tau and apoE were dichotomized as low or high based on the cohort median. In addition, higher p-tau in the FA fraction was associated with reduction of CLDN5 (*P* < .001; Table 3), suggesting that aggregated p-tau may also contribute to the decrease of tight junction marker. No evidence of interaction between insoluble p-tau and apoE (TBS; *P* = .72) or apoE (TX; *P* = .87) was observed with regard to association with CLDN5, respectively.

### 3.3 | Tight junction protein levels are not influenced by CAA severity in AD brains

To determine the relationship between cerebrovascular tight junction damage and the severity of CAA in AD, the associations of CLDN5 and OCLN with CAA score were examined. Averaged CAA score was not associated with either CLDN5 or OCLN levels in the temporal cortex of AD (Table 4), and this lack of association was consistent when stratifying by sex and *APOE ε*4 (all *P* ≥ .22). Similarly, we did not observe an association with CAA score in the superior temporal cortex for either CLDN5 or OCLN (all *P* ≥ .081). These results indicate that the reduction of tight junction proteins, which suggests a compromised BBB integrity in the temporal cortex region of AD brain, is likely independent of overall CAA pathology in the cerebrovasculature.

### 3.4 | Amount of tau does not associate with CAA severity in AD brains

To investigate the impacts of AD-related molecules on another vascular pathology, we assessed their associations with the CAA score. While tau levels in each fraction were not significantly associated with CAA severity after correcting for multiple testing, we found that the greater CAA score was associated with a higher A*β*40 and A*β*40/A*β*42 ratio (all *P* < .001) in all fractions as well as with higher apoE levels in TX and FA fractions in multivariable analysis (all *P* < .001; Table 4). Consistent with our previous study using AD subjects with severe CAA or without CAA,^21^ we confirmed that male sex and the *APOE ε*4 allele were significantly associated with a greater CAA score in multivariable analysis (all *P* ≤ .002 for analysis of all patients) in this larger AD cohort with a broad spectrum of CAA scores (Table S4; Figure S1 in supporting information).

We also examined the associations of sex and *APOE ε*4 with AD-related molecules. In multivariable analysis, *APOE ε*4 was significantly associated with lower soluble apoE but higher insoluble apoE (all *P* < .001), whereas other AD-related molecules were not associated with male sex or *APOE ε*4 (all *P* ≥ .012, *P* < .0083 considered significant after multiple testing adjustment).

## 4 | DISCUSSION

The cerebrovascular pathologies of AD, including CAA and arteriosclerosis/fibrolipohyalinosis in small blood vessels, have been shown to correlate with cognitive decline during disease progression.^38^ CAA resulting from the deposition of A*β* in vascular walls likely compromises the integrity of the neurovascular units in AD. In addition, BBB leakage detected by contrast-enhanced MRI has been shown to be a principal mechanism contributing to WMHs in cSVD.^15^ The BBB plays a critical role in maintaining CNS homeostasis; thus, the disturbance of BBB function is increasingly recognized as a potential contributor to the pathogeneses of various neurological diseases, including AD.^18^ Capillary endothelial cells form the BBB, in which the intramembrane proteins including CLDN5 and OCLN at tight junction and cadherin at adherens junction maintain the continuous endothelial connections with highly regulated physical and metabolic barrier integrity.^17,39^ Thus, understanding the mechanisms by which BBB function is modulated will provide novel insights regarding potential pathogenic pathways for the vascular dysfunction in AD. Our current study investigated how AD-related molecules, *APOE* isoforms, and sex impact cerebrovascular damages, and whether CAA and BBB integrity interact in AD *post mortem* brains with comorbid pathologies. We biochemically measured the amounts of key tight junction proteins, CLDN5 and OCLN, in the temporal cortex of a large *post mortem* brain cohort (N = 469) of neuropathologically confirmed AD cases. We demonstrate that the reduction of tight junction proteins, at least in the temporal cortex, may be an independent event from CAA formation in AD (Figure S2 in supporting information). In addition, although A*β*40 levels were strongly correlated with CAA severity, we did not find an association between A*β*40 and the tight junction protein levels. While the disturbance of A*β*40 elimination through interstitial fluid drainage pathway along basement membranes in vascular smooth muscle cell layers likely triggers CAA pathogenesis,^40,41^ our results imply that BBB damage is not directly linked to the pathogenic mechanism of CAA in the temporal cortex. In contrast, IgG leakage in the frontotemporal and parietooccipital cortex is likely increased in CAA cases compared with cognitively normal controls,^42^ although CAA-related cerebral microbleeds may cause the infiltration of circulating molecules into the bran independently of BBB integrity. Previous studies have shown that A*β* exerts profound effects on the regulation of the cerebral blood flow.^43^ As A*β* also deposits on brain capillaries in a subset of CAA,^44^ capillary CAA may influence cerebral blood flow reduction which in turn correlates with BBB impairment in cSVD.^45^ Future studies are needed to further determine the relationship between capillary and non-capillary CAA subtypes and BBB dysregulation.

We previously reported a marked reduction of tight junction proteins in AD brains compared to those in age-matched normal aging and pathological aging subjects.^19^ As A*β*42 levels were significantly higher in AD and pathological aging brains than normal aging cases, this phenomenon may contribute to the negative correlation observed between A*β*42 and tight junction.^19^ Such an interaction was not observed in current study when these molecules were compared among AD brains with abundant A*β* pathology. In addition, our previous study identified an association between CAA and tight junction protein levels when examining a combined series of normal aging, pathological aging, and AD subjects,^19^ whereas such association was not observed among AD brains in current study.

Importantly, our study has clearly shown that soluble tau causatively or consequently contributes to the reduction of tight junction protein levels. Consistent with this finding, a brain imaging study in VCID patients revealed that WMH volume, numbers of lacunes and microbleeds are associated with tau accumulation in the inferior temporal and medial temporal regions shown by higher uptake of ^18^F-AV1451 positron emission tomography (PET) tracer.^28^ Additionally, the P-glycoprotein transport system at the BBB was disturbed in progressive supranuclear palsy, a neurodegenerative disease with primary tauopathy.^46^ Furthermore, human P301L tau has been shown to cause BBB breakdown in doxycycline-regulated rTg4510 mice.^47^ Interestingly, PAI-1, vascular endothelial growth factor A (VEGFA), uPA, and MMP9, which are involved in the vascular remodeling pathway, are upregulated in cerebrovascular endothelial cells isolated from rTg4510 tauopathy model mice. These lines of evidence indicate that tau has a specific role in regulating the cerebrovascular system. We observed a positive correlation between tight junction markers and soluble p-tau (TX) whose reduction might be an indication of p-tau aggregation, leading to an increase of insoluble tau (FA). Alternatively, tau may induce blood vessel abnormalities and aberrant angiogenesis. Whether soluble p-tau induces vascular remodeling in AD brains requires further investigation.

Excess accumulation of tau may disturb the vascular remodeling pathway, although it remains to be elucidated if soluble tau directly interacts with tight junction proteins at the BBB or indirectly by perturbing specific signaling pathways. Alternatively, it is also possible that cerebrovascular damage causes neuronal injuries which aggravate the accumulation of tau. Certainly, the vascular risk quantified by the office-based Framingham Heart Study cardiovascular disease risk score according to risk factors including anti-hypertensive treatment, systolic blood pressure, body mass index, history of diabetes, and current cigarette smoking status, and so on, has been shown to correlate with increased cortical tau accumulation in healthy older individuals.^27^ Thus, BBB dysregulation could exacerbate tau accumulation, which might further compromise BBB function, triggering a vicious cycle between tauopathy and BBB leakage in AD. Indeed, we found that the higher insoluble p-tau was associated with lower CLDN5 levels, suggesting that aggregation of p-tau or tau pathology may contribute to the impairment of BBB integrity in AD. Consistent with this notion, the immunoreactivity patterns of tight junction proteins are shown to be discontinuous or absent in perivascular regions with p-tau deposition in patients with chronic traumatic encephalopathy (CTE), indicating that perivascular p-tau accumulation is accompanied by BBB disruption.^48^ Given that conformation of tau aggregates around blood vessels in CTE is likely different from that in the brain parenchyma of AD,^49^ future studies focusing on the structures of tau filaments in AD may allow us to dissect the mechanisms of tau-mediated BBB damage and BBB disruption-associated tauopathy.

Our results also indicate that apoE is involved in both CAA formation and BBB damage in AD. While apoE is possibly co-aggregated with A*β* in CAA, higher apoE levels were also associated with reduced tight junction proteins independently of tau accumulation. An immuno-histochemical study found that apoE expression positively correlates with VEGF and endothelial nitric oxide synthase (eNOS) expression in the capillaries of AD brains.^50^ Because VEGF suppresses CLDN5 and OCLN expression^51^ and eNOS increases BBB permeability,^52^ it is possible that apoE compromises endothelial integrity by modulating those factors. As insoluble A*β*42 is positively correlated with OCLN levels, A*β* may exhibit a protective role in BBB damage by antagonizing the apoE effect. Although further studies are required, it is also possible that apoE levels are increased as a consequence of BBB breakdown. Endothelial dysregulation may result in leakage of peripheral apoE, compromised apoE clearance at the perivascular region, or an increase in apoE production by activated glial cells. *APOE ε*4 status did not influence tight junction protein levels in our study though BBB dysfunction was observed in apoE4-targeted replacement (TR) mice.^53–55^ Because *APOE ε*4 exacerbates the impact of vascular risk factors on white matter damage,^56^*APOE ε*4 may complicate the symptoms of BBB dysregulation by modulating basement membrane components and pericyte/astrocyte properties rather than directly disrupting tight junction components. Alternatively, the effect of *APOE ε*4 on BBB function may be diminished in the presence of a high concentration of tau or A*β*.

In summary, our study has revealed the respective contributions of tau and A*β*40 to tight junction reduction, and CAA formation in AD, while apoE was associated with both cerebrovascular phenotypes. One limitation of our study is that the biochemical investigations were performed in the temporal cortex, which is one of the most AD neuropathology-affected regions, but not the most CAA-abundant region. Future studies will investigate the association of BBB integrity with tau, A*β*, and other AD-related molecules in multiple brain regions. Although our study has limitations in terms of addressing only one aspect of BBB damage in one brain region of end-stage AD cases, we demonstrated that BBB impairment and CAA formation could be attributed to different neuropathologic mechanisms in AD. These findings generate potentially novel hypotheses regarding mechanisms concerning how these AD-related molecules impact cerebral vascular dysregulation in AD pathogenesis and warrants further investigation. In addition, the levels of tight junction proteins were normalized by the levels of endothelial cell marker CD31 to minimize the potential influence of different cerebrovascular vessel density among samples.^19^ However, as CD31 expression may be affected by angiogenesis and immune cell extravasation during neuroinflammation,^57,58^ how tau and apoE impact the cerebrovascular endothelial integrity requires further investigations through neuropathological and functional studies to compensate the limitation of our biochemical approaches. Because the cerebrovascular pathologies possibly cause cognitive impairment in an independent manner in AD, developing a combination therapy to ameliorate non–CAA-related BBB damage and CAA might be critically important to treat this complex disease. Future longitudinal studies monitoring tau/A*β* accumulation, cSVD, BBB leakage, and CAA formation comprehensively in *ante mortem* cohorts should provide further clues as to how the cerebrovascular system can be targeted for therapy in VCID and AD. While we did not observe an association between tau levels and CAA severity, it has been reported that tau predominantly mediates cerebrovascular damages including arterial smooth muscle loss, elastin degradation, and stiffness prior to CAA formation in early-stage AD with Braak I-II.^59^ Therefore, early interventions with tau-targeted therapy might be potentially beneficial in preventing the progression of VCID and AD.

## Supplementary Material

supplement

## Figures and Tables

**TABLE 1:** Subject characteristics of Alzheimer’s disease (AD) brain samples

	*APOE ε4* non-carrier (N = 158)		*APOE ε4* carrier (n = 311)		
	Female (n = 79)	Male (n = 79)	Female (n = 166)	Male (n = 145)	Overall (n = 469)
Age at death (years)	82 (57, 98)	78 (55, 93)	83 (55, 100)	81 (57, 95)	82 (55, 100)
Braak stage					
IV	9 (11.4%)	14 (17.7%)	22 (13.3%)	26 (17.9%)	71 (15.1%)
V	27 (34.2%)	33 (41.8%)	39 (23.5%)	44 (30.3%)	143 (30.5%)
VI	43 (54.4%)	32 (40.5%)	105 (63.3%)	75 (51.7%)	255 (54.4%)
Thal phase					
2	1 (1.3%)	1 (1.3%)	1 (0.6%)	0 (0.0%)	3 (0.6%)
3	6 (7.6%)	11 (14.1%)	8 (4.8%)	7 (4.8%)	32 (6.8%)
4	4 (5.1%)	6 (7.7%)	17 (10.2%)	10 (6.9%)	37 (7.9%)
5	68 (86.1%)	60 (76.9%)	140 (84.3%)	128 (88.3%)	396 (84.6%)
CAA score	0.40 (0, 2.60)	0.50 (0, 2.20)	0.80 (0, 4.00)	0.80 (0, 3.88)	0.80 (0, 4.00)

The sample median (minimum, maximum) is given for continuous variables. Information was unavailable for Thal phase (n = 1).

Abbreviation: APOE, apolipoprotein E; CAA, cerebral amyloid angiopathy

**TABLE 2:** Pair-wise correlations among Alzheimer’s disease (AD)-related molecules and tight junction proteins in the temporal cortex of AD cases

				Spearman’s r (*P*-value)												
	apoETX	apoEFA		A*β*40 TX	A*β*40FA	A*β*42 TBS	A*β*42 TX	A*β*42FA	T-tau TBS	T-tau TX	T-tau FA	p-tau TBS	p-tau TX	p-tau FA	CLDN5 TX	OCLN TX
**ApoE** TBS	**0.45 (<0.001)**	0.01 (0.85)	−0.07 (0.11)	−0.06 (0.19)	**−0.15 (0.002)**	**0.14 (0.003)**	0.01 (0.76)	**−0.09 (0.046)**	**0.26 (<0.001)**	**0.23 (<0.001)**	**0.18 (<0.001)**	**0.26 (<0.001)**	−0.04 (0.38)	−0.01 (0.83)	**−0.29 (<0.001)**	**−0.26 (<0.001)**
TX	1.00	**0.27 (<0.001)**	**0.12 (0.011)**	**0.33 (<.0001)**	0.03 (0.58)	**−0.12 (0.008)**	0.01 (0.77)	−0.07 (0.15)	**0.34 (<0.001)**	0.01 (0.87)	**−0.15 (0.001)**	0.04 (0.44)	**−0.13 (0.004)**	−0.07 (0.15)	**−0.18 (<0.001)**	**−0.31 (<0.001)**
FA	-	1.00	**0.31 (<0.001)**	**0.37 (<0.001)**	**0.48 (<0.001)**	0.07 (0.11)	0.08 (0.068)	0.08 (0.095)	**−0.10 (0.027)**	**−0.18 (<0.001)**	**0.24 (<0.001)**	0.01 (0.72)	**0.29 (<0.001)**	**0.45 (<0.001)**	**−0.17 (<0.001)**	−0.06 (0.21)
**A*β*40** TBS	-	-	1.00	**0.52 (<0.001)**	**0.63 (<0.001)**	**0.29 (<0.001)**	**0.22 (<0.001)**	**0.15 (0.001)**	0.06 (0.17)	0.08 (0.100)	0.07 (0.13)	−0.06 (0.16)	0.00 (0.99)	0.09 (0.059)	−0.04 (0.35)	0.01 (0.83)
TX	-	-	-	1.00	**0.55 (<0.001)**	**0.13 (0.004)**	**0.25 (<0.001)**	0.07 (0.15)	0.03 (0.51)	−0.04 (0.44)	−0.08 (0.069)	−0.04 (0.38)	0.04 (0.40)	0.05 (0.30)	−0.04 (0.41)	**−0.10 (0.030)**
FA	-	-	-	-	1.00	**0.21 (<0.001)**	**0.19 (<0.001)**	**0.23 (<0.001)**	**−0.12 (0.009)**	−0.06 (0.22)	**0.12 (0.013)**	−0.07 (0.13)	**0.21 (<0.001)**	**0.25 (<0.001)**	0.02 (0.72)	**0.09 (0.042)**
**A*β*42** TBS	-	-	-	-	-	1.00	**0.66 (<0.001)**	**0.41 (<0.001)**	**0.23 (<0.001)**	**0.44 (<0.001)**	**0.39 (<0.001)**	**0.34 (<0.001)**	**0.11 (0.022)**	**0.19 (<0.001)**	**−0.17 (<0.001)**	**0.18 (<0.001)**
TX	-	-	-	-	-	-	1.00	**0.53 (<0.001)**	**0.20 (<0.001)**	**0.39 (<0.001)**	**0.29 (<0.001)**	**0.27 (<0.001)**	**0.22 (<0.001)**	**0.17 (<0.001)**	−0.05 (0.31)	**0.26 (<0.001)**
FA	-	-	-	-	-	-	-	1.00	0.05 (0.25)	**0.18 (<0.001)**	0.22 (<0.001)	0.08 (0.10)	**0.21 (<0.001)**	**0.17 (<0.001)**	0.04 (0.40)	**0.32 (<0.001)**
**T-tau** TBS	-	-	-	-	-	-	-	-	1.00	**0.60 (<0.001)**	0.07 (0.14)	**0.09 (0.045)**	**−0.21 (<0.001)**	**−0.38 (<0.001)**	**−0.26 (<0.001)**	**−0.15 (0.001)**
TX	-	-	-	-	-	-	-	-	-	1.00	**0.29 (<0.001)**	**0.16 (<0.001)**	**−0.54 (<0.001)**	**−0.29 (<0.001)**	**−0.18 (<0.001)**	**0.17 (<0.001)**
FA	-	-	-	-	-	-	-	-	-	-	1.00	**0.14 (0.002)**	**0.14 (0.002)**	**0.39 (<0.001)**	**−0.13 (0.005)**	**0.18 (<0.001)**
**p-Tau** TBS	-	-	-	-	-	-	-	-	-	-	-	1.00	**0.24 (<0.001)**	0.03 (0.45)	−0.03 (0.49)	**0.15 (<0.001)**
TX	-	-	-	-	-	-	-	-	-	-	-	-	1.00	**0.61 (<0.001)**	0.07 (0.15)	**0.24 (<0.001)**
FA	-	-	-	-	-	-	-	-	-	-	-	-	-	1.00	**−0.15 (0.002)**	0.06 (0.19)
**CLDN5** TX															1.00	**0.48 (<0.001)**
**OCLN** TX	-	-	-	-	-	-	-	-	-	-	-	-	-		-	1.00

*P*-values <.05 were considered statistically significant in this exploratory correlation analysis; statistically significant correlations are shown in bold.

Abbreviation: APOE, apolipoprotein E; CAA, cerebral amyloid angiopathy; FA, formic acid; TBS, Tris-buffered saline; TX, 1% Triton X-100

**TABLE 3:** Associations of tight junction proteins with Alzheimer’s disease (AD)-related molecules (apoE, A*β*40, A*β*42, and total tau)

	Association with CLDN5 TX/CD31 TX				Association with OCLN TX/CD31 TX			
	Unadjusted analysis		Adjusting for age at death, sex, CAA score, number of APOE *ε*4 alleles, Braak stage, and Thai phasellnadjusted analysis		Unadjusted analysis		Adjusting for age at death, sex, CAA score, number of APOE *ε*4 alleles, Braak stage, and Thai phase	
Variable	Regression coefficient (95% CI)	*P*-value	Regression coefficient (95% CI)	*P*-value	Regression coefficient (95% CI)	*P*-value	Regression coefficient (95% CI)	*P*-value
A*β*40 TBS	−0.03 (−0.13, 0.06)	.51	−0.03 (−0.14, 0.07)	.52	0.00 (−0.02, 0.03)	.67	0.01 (−0.01, 0.03)	.34
A*β*40 TX	−0.04 (−0.14, 0.05)	.36	−0.06 (−0.16, 0.05)	.32	−0.02 (−0.04, 0.00)	.11	−0.02 (−0.04, 0.01)	.20
A*β*40 FA	0.01 (−0.09, 0.10)	.84	0.02 (−0.09, 0.14)	.68	0.02 (0.00, 0.04)	.086	**0.04 (0.01, 0.07)**	**.002**
A*β*42 TBS	**−0.23 (−0.32, −0.13)**	**<.001**	**−0.22 (−0.32, −0.13)**	**<.001**	0.03 (0.01, 0.05)	.004	0.03 (0.01, 0.05)	.0033
A*β*42 TX	−0.12 (−0.21, −0.02)	.017	−0.10 (−0.20, −0.01)	.031	**0.04 (0.02, 0.06)**	**<.001**	**0.05 (0.03, 0.07)**	**<.001**
A*β*42 FA	−0.04 (−0.13, 0.06)	.41	−0.04 (−0.14, 0.05)	.39	**0.06 (0.04, 0.08)**	**<.001**	**0.07 (0.05, 0.09)**	**<.001**
A*β*40/42 TBS	0.06 (−0.04, 0.15)	.25	0.07 (−0.04, 0.17)	.20	−0.01 (−0.03, 0.01)	.51	0.00 (−0.02, 0.02)	.82
A*β*40/42 TX	0.02 (−0.07, 0.12)	.64	0.02 (−0.08, 0.13)	.67	**−0.04 (−0.06, −0.02)**	**<.001**	**−0.05 (−0.07, −0.03)**	**<.001**
A*β*40/42 FA	0.03 (−0.07, 0.12)	.58	0.05 (−0.06, 0.17)	.39	−0.01 (−0.03, 0.01)	.41	0.00 (−0.03, 0.02)	.79
Total tau TBS	**−0.38 (−0.47, −0.30)**	**<.001**	**−0.43 (−0.52, −0.34)**	**<.001**	**−0.04 (−0.06, −0.02)**	**<.001**	**−0.05 (−0.07, −0.03)**	**<.001**
Total tau TX	**−0.29 (−0.38, −0.20)**	**<.001**	**−0.32 (−0.41, −0.22)**	**<.001**	0.02 (0.00, 0.04)	.031	0.02 (0.00, 0.04)	.13
Total tau FA	−0.13 (−0.22, −0.03)	.008	−0.13 (−0.22, −0.03)	.009	0.03 (0.01, 0.05)	.005	0.03 (0.01, 0.05)	.007
p-tau TBS	−0.07 (−0.17, 0.02)	.11	−0.08 (−0.17, 0.02)	.12	0.02 (−0.00, 0.04)	.077	0.02 (0.00, 0.04)	.053
p-tau TX	0.14 (0.04, 0.23)	.004	**0.17 (0.07, 0.27)**	**.001**	**0.06 (0.04, 0.08)**	**<.001**	**0.08 (0.06, 0.10)**	**<.001**
p-tau FA	**−0.17 (−0.27, −0.08)**	**<.001**	**−0.20 (−0.30, −0.09)**	**<.001**	0.01 (−0.01, 0.03)	.51	0.02 (0.00, 0.05)	.048
apoE TBS	**−0.32 (−0.41, −0.23)**	**<.001**	**−0.38 (−0.47, −0.28)**	**<.001**	**−0.06 (−0.08, −0.04)**	**<.001**	**−0.07 (−0.09, −0.05)**	**<.001**
apoE TX	**−0.18 (−0.27, −0.09)**	**<.001**	**−0.21 (−0.30, −0.11)**	**<.001**	**−0.06 (−0.08, −0.04)**	**<.001**	**−0.07 (−0.09, −0.05)**	**<.001**
apoE FA	**−0.21 (−0.30, −0.11)**	**<.001**	**−0.27 (−0.37, −0.16)**	**<.001**	−0.01 (−0.03, 0.01)	.18	−0.01 (−0.03, 0.02)	.61

Regression coefficients, 95% CIs, and *P*-values result from linear regression models, where CLDN5 TX/CD31 TX was considered on the original scale and OCLN TX/CD31 TX was considered on the square root scale. Regression coefficients are interpreted as the change in mean outcome level (CLDN5 TX/CD31 TX or OCLN TX/CD31 TX, on the aforementioned scales) corresponding to each 1-standard-deviation increase in the given variable (on the untransformed, square root transformed, or natural logarithm transformed scale). *P*-values <.0028 were considered statistically significant after applying a Bonferroni correction for multiple testing separately for each tight junction protein measure; statistically significant associations are shown in bold.

Abbreviation: APOE, apolipoprotein E; CAA, cerebral amyloid angiopathy; CI, confidence interval; CLDN5, claudin-5; FA, formic acid; OCLN, occludin; TBS, Tris-buffered saline; TX, 1% Triton X-100

**TABLE 4:** Associations of tight junction markers and Alzheimer’s disease (AD)-related molecules with cerebrovascular pathologies including CAA score

	Association with CAA score			
	Unadjusted analysis		Adjusting for age at death, sex, number of *APOE ε*4 alleles, Braak stage, and Thal phase	
Variable	Regression coefficient (95% CI)	*P*-value	Regression coefficient (95% CI)	*P*-value
A*β*40 TBS	**0.16 (0.13, 0.20)**	**<.001**	**0.13 (0.10, 0.16)**	**<.001**
A*β*40 TX	**0.20 (0.16, 0.23)**	**<.001**	**0.17 (0.13, 0.20)**	**<.001**
A*β*40 FA	**0.24 (0.21, 0.27)**	**<.001**	**0.21 (0.17, 0.24)**	**<.001**
A*β*42 TBS	0.04 (0.00, 0.07)	.068	0.03 (0.00, 0.06)	.085
A*β*42 TX	0.03 (−0.01, 0.06)	.19	0.01 (−0.02, 0.05)	.47
A*β*42 FA	0.05 (0.01, 0.09)	.007	**0.06 (0.02, 0.09)**	**.001**
A*β*40/A*β*42 TBS	**0.16 (0.12, 0.19)**	**<0.001**	**0.13 (0.09, 0.16)**	**<0.001**
A*β*40/A*β*42 TX	**0.18 (0.15, 0.22)**	**<.001**	**0.15 (0.12, 0.19)**	**<.001**
A*β*40/A*β*42 FA	**0.22 (0.19, 0.26)**	**<.001**	**0.19 (0.16, 0.22)**	**<.001**
Total tau TBS	−0.04 (−0.08, 0.00)	.043	−0.04 (−0.07, 0.00)	.033
Total tau TX	−0.04 (−0.08, −0.01)	.023	−0.04 (−0.07, 0.00)	.045
Total tau FA	−0.03 (−0.07, 0.01)	.12	−0.03 (−0.07, 0.00)	.074
p-tau TBS	−0.02 (−0.06, 0.02)	.25	−0.02 (−0.06, 0.01)	.17
p-tau TX	0.04 (0.00, 0.08)	.044	0.04 (0.00, 0.07)	.047
p-tau FA	0.06 (0.02, 0.09)	.004	0.04 (0.00, 0.08)	.048
apoE TBS	−0.05 (−0.09, −0.01)	.008	0.00 (−0.03, 0.04)	.88
apoE TX	0.04 (0.00, 0.08)	.027	**0.07 (0.03, 0.10)**	**<.001**
apoE FA	**0.15 (0.11, 0.18)**	**<.001**	**0.11 (0.07, 0.14)**	**<.001**
CLDN5 TX/CD31 TX	0.00 (−0.04, 0.04)	.98	0.01 (−0.02, 0.05)	.42
OCLN TX/CD31 TX	−0.01 (−0.05, 0.03)	.51	0.00 (−0.03, 0.04)	.86

Regression coefficients, 95% CIs, and *P*-values result from linear regression models, where CAA score was considered on the square root scale. Regression coefficients are interpreted as the change in mean CAA score (on the square root scale) corresponding to each 1-standard-deviation increase in the given variable (on the untransformed, square root transformed, or natural logarithm transformed scale). *P*-values <.0025 were considered statistically significant after applying a Bonferroni correction for multiple testing separately for each tight junction protein measure; statistically significant associations are shown in bold.

Abbreviation: APOE, apolipoprotein E; CAA, cerebral amyloid angiopathy; CI, confidence interval; CLDN5, claudin-5; FA, formic acid; OCLN, occludin; TBS, Tris-buffered saline; TX, 1% Triton X-100

## RESEARCH IN CONTEXT

1. Systematic review: The authors reviewed the literature using PubMed, Google, Research Gate, and through meeting abstracts and presentations. While the way different neuropathologic components interact to impact vascular integrity in human Alzheimer’s disease (AD) has not been systematically studied, several publications implicate the roles of vascular dysfunctions in AD. The relevant citations are appropriately cited.
2. Interpretation: Using a large, neuropathologically confirmed human AD cohort representing the broad spectrum of AD population, we report that higher levels of soluble tau, insoluble p-tau, and apolipoprotein E (apoE), but not CAA severity, were independently correlated with lower endothelial tight junction proteins. These results indicate that blood-brain barrier (BBB) damage and cerebral amyloid angiopathy (CAA) pathology in AD brains may be the result of distinct neuropathologic mechanisms.
3. Future directions: Our findings refine the molecular mechanisms regarding respective contributions of tau, amyloid beta, and apoE to cerebrovascular integrity and pathologies which provide novel insights into the potential therapeutic strategy targeting tau-mediated BBB dysfunction in AD.
