# Supplementary material for: Tau and apolipoprotein E modulate cerebrovascular tight junction integrity independent of cerebral amyloid angiopathy in Alzheimer’s disease
Source: Alzheimers Dement. Author manuscript; Available in PMC 2021 May 7. (PMC8103951; doi:10.1002/alz.12104)
Supplement: supplement [file NIHMS1693748-supplement-supplement.pdf]

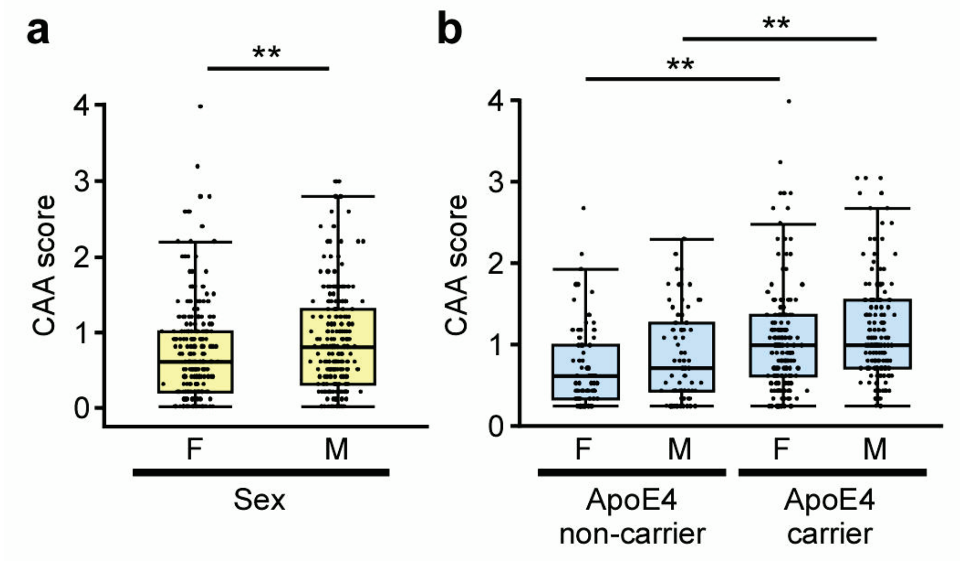

Supplemental Fig. 1. Impact of sex and *APOE4* status on CAA severity.

Boxplots of CAA score according to sex and *APOE4* status were shown. F, female; M; male.**, P <0.01.

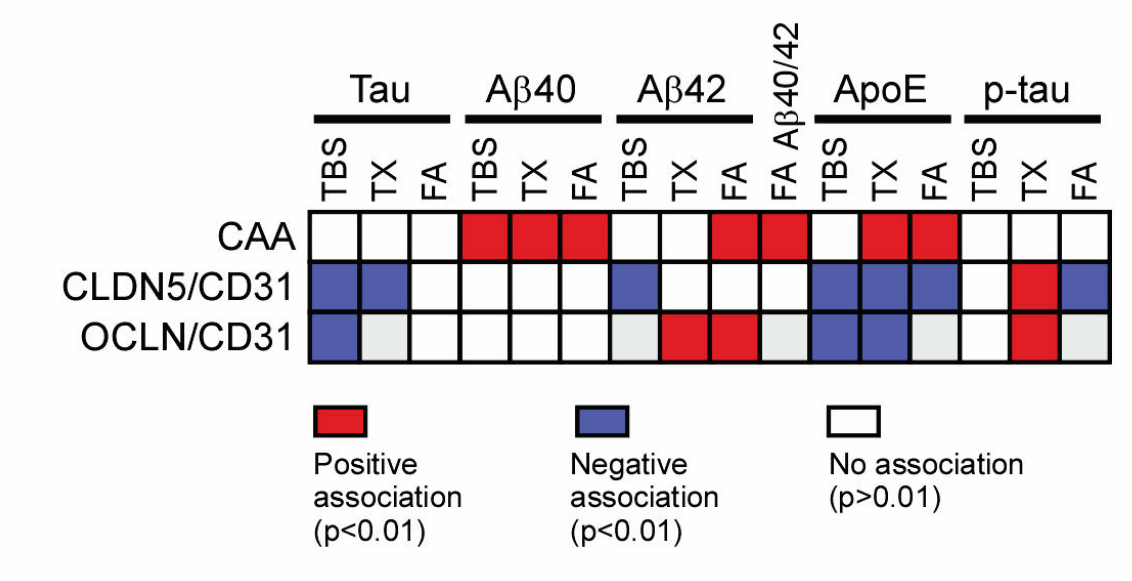

Supplemental Fig. 2. Correlations of AD-related molecules with CAA score and tight junction protein levels.

Heat-map summarizing statistically significant correlations between CAA and AD-related molecules (tau, Aβ40, Aβ42, Aβ40/42 ratio, apoE and p-Tau) in different fractions or between tight junction proteins and AD-related molecules are shown.

Supplemental Table 1: Linearity-of-dilution and Spike-and-recovery assessments for ELISAs.

ELISA linearity-of-dilution results for apoE. in different fractions.

Sample	Dilution factor (DF)	Observed x DF (ng/ml)	Expected (ng/ml)	Recovery (%)
ApoE (TBS)	Neat	3753.8	3753.8	100
	1:2	3438.9		91.6
	1:4	2774.6		73.9
	1:8	3078.1		82.0
ApoE (TBSX)	Neat	4900.4	4900.4	100.0
	1:2	5044.9		102.9
	1:4	5821.6		118.8
	1:8	5513.4		112.5
ApoE (FA)	Neat	78.9	78.9	100.0
	1:2	93.3		118.2
	1:4	93.5		118.5
	1:8	105.8		134.0

Spike and recovery analysis for apoE in different fractions.

Sample	Spike (ng/ml)	Observed (ng/ml)	Expected (ng/ml)	Recovery (%)
ApoE (TBS)	Neat	5.7	5.7	100.0
	10	12.2	15.7	77.5
	2.5	6.7	8.2	81.6
	0.625	5.6	6.4	88.5
ApoE (TBSX)	Neat	8.4	8.4	100.0
	10	15.2	18.4	82.8
	2.5	10.0	10.9	91.4
	0.625	9.5	9.0	105.6
ApoE (FA)	Neat	4.7	4.7	100.0
	10	12.7	14.7	86.5
	2.5	7.2	7.2	100.3
	0.625	6.0	5.3	112.4

ELISA linearity-of-dilution results for tau in different fractions.

Sample	Dilution factor (DF)	Observed x DF (ng/ml)	Expected (ng/ml)	Recovery (%)
Tau (TBS)	Neat	1082.7	1082.7	100.0
	1:2	1279.4		118.2
	1:4	1297.3		119.8
	1:8	1170.6		108.1
Tau (TBSX)	Neat	1947.0	1947.0	100.0
	1:2	1624.5		83.4
	1:4	1750.5		89.9
Tau (FA)	Neat	76.8	76.8	100.0
	1:2	81.5		106.2
	1:4	83.5		108.7
	1:8	86.7		113.0

Spike and recovery analysis for tau in different fractions.

Sample	Spike (ng/ml)	Observed (ng/ml)	Expected (ng/ml)	Recovery (%)
Tau (TBS)	Neat	2.8	2.8	100.0
	3.2	5.4	6.0	89.7
	2.4	4.8	5.2	92.3
	1.6	4.2	4.4	95.6
Tau (TBSX)	Neat	1.4	1.4	100.0
	3.2	4.4	4.6	94.3
	2.4	3.6	3.8	95.1
	1.6	3.2	3.0	104.2
Tau (FA)	Neat	3.0	3.0	100.0
	3.2	5.7	6.2	90.5
	2.4	4.7	5.4	86.8
	1.6	4.1	4.6	87.8

Supplemental Table 1: Assessments for Aβ ELISAs.

ELISA linearity-of-dilution results for Aβ40 in different fractions.

Sample	Dilution factor (DF)	Observed x DF (ng/ml)	Expected (ng/ml)	Recovery (%)
Aβ40 (TBS)	Neat	7.1	7.1	100.0
	1:2	7.5		105.0
	1:4	7.9		110.4
Aβ40 (TBSX)	Neat	10.3	10.3	100.0
	1:2	11.5		111.8
	1:4	11.8		114.1
Aβ40 (FA)	Neat	442.4	442.4	100.0
	1:2	402.0		90.9
	1:4	425.8		96.3

Spike and recovery analysis for Aβ40 in different fractions.

Sample	Spike (pg/ml))	Observed (pg/ml)	Expected (pg/ml)	Recovery (%)
Aβ40 (TBS)	Neat	102.0	102.0	100.0
	800	1057.4	902.0	117.2
	400	468.6	502.0	93.4
	200	281.1	302.0	93.1
Aβ40 (TBSX)	Neat	730.6	730.6	100.0
	800	1886.8	1530.6	123.3
	400	1136.1	1130.6	100.5
	200	761.8	930.6	81.9
Aβ40 (FA)	Neat	425.0	425.0	100.0
	800	1468.6	1225.0	119.9
	400	696.1	825.0	84.4
	200	516.1	625.0	82.6

ELISA linearity-of-dilution results for Aβ42 in different fractions.

Sample	Dilution factor (DF)	Observed x DF (ng/ml)	Expected (ng/ml)	Recovery (%)
Aβ42 (TBS)	Neat	3.5	3.5	100.0
	1:2	3.6		101.7
	1:4	3.5		100.5
Aβ42 (TBSX)	Neat	4.2	4.2	100.0
	1:2	4.0		95.2
	1:4	5.4		129.8
Aβ42 (FA)	Neat	392.1	392.1	100.0
	1:2	380.2		97.0
	1:4	376.3		96.0

Spike and recovery analysis for Aβ42 in different fractions.

Sample	Spike (pg/ml)	Observed (pg/ml)	Expected (pg/ml)	Recovery (%)
Aβ42 (TBS)	Neat	182.4	182.4	100.0
	400	581.7	582.4	99.9
	200	347.4	382.4	90.8
	100	244.6	282.4	86.6
Aβ42 (TBSX)	Neat	946.0	946.0	100.0
	400	1606.7	1346.0	119.4
	200	1312.4	1146.0	114.5
	100	1094.6	1046.0	104.6
Aβ42 (FA)	Neat	768.9	768.9	100.0
	400	1126.0	1168.9	96.3
	200	933.1	968.9	96.3
	100	843.9	868.9	97.1

Supplemental Table 1: Assessments for Tight junction ELISAs.

ELISA linearity-of-dilution results for CLDN5 in different fractions.

Sample	Dilution factor (DF)	Observed x DF (ng/ml)	Expected (ng/ml)	Recovery (%)
CLDN5 (TBSX)	Neat	1403.6	1403.6	100.0
	1:2	1419.2		101.1
	1:4	1494.2		106.5
	1:8	1400.3		99.8

Spike and recovery analysis for CLDN5 in different fractions.

Sample	Spike (ng/ml)	Observed (ng/ml)	Expected (ng/ml)	Recovery (%)
CLDN5 (TBSX)	Neat	17.4	17.4	100.0
	100	98.8	117.4	84.2
	50	68.9	67.4	102.1
	12.5	28.3	29.9	94.5

ELISA linearity-of-dilution results for CD31 in TBSX fraction.

Sample	Dilution factor (DF)	Observed x DF (ng/ml)	Expected (ng/ml)	Recovery (%)
OCLN (TBSX)	Neat	1035.4	1035.4	100.0
	1:2	1114.4		107.6
	1:4	959.9		92.7
	1:8	1008.1		97.4

Spike and recovery analysis for CD31 in TBSX fraction.

Sample	Neat (ng/ml)	Spike level (ng/ml)	Observed (ng/ml)	Recovery (%)
OCLN (TBSX)	129.4	0	129.4	100.0
	129.4	60.0	189.1	99.8
	69.7	30.0	93.0	93.3
	30.0	15.0	41.9	93.1

ELISA linearity-of-dilution results for CD31 in TBSX fraction.

Sample	Dilution factor (DF)	Observed x DF (ng/ml)	Expected (ng/ml)	Recovery (%)
CD31 (TBSX)	Neat	68.5	68.5	100.0
	1:2	77.3		112.8
	1:4	80.8		117.9
	1:8	91.2		133.2

Spike and recovery analysis for CD31 in TBSX fraction.

Sample	Neat (ng/ml)	Spike level (ng/ml)	Observed (ng/ml)	Recovery (%)
CD31 (TBSX)	8.5	0	8.5	100.0
	8.5	2.0	10.2	97.0
	4.8	1.0	6.3	108.8
	2.8	0.5	3.7	111.1

Supplemental Table 2: the intra- and inter-assay coefficients of variation on the ELISA measurements.

	Aβ40			***Aβ42***			***Tau***		
	TBS	TBSX	FA	TBS	TBSX	FA	TBS	TBSX	FA
**Intra-assay (%CV)**	6.50	5.93	5.67	4.18	3.52	5.88	5.85	4.40	3.70
**Inter-assay (%CV)**	5.31	8.40	5.32	6.21	5.73	5.80	8.58	9.40	9.20
	CLDN5	***OCLN***	***CD31***	***ApoE***			***p-Tau***		
	TBSX	TBSX	TBSX	TBS	TBSX	FA	TBS	TBSX	FA
**Intra-assay (%CV)**	5.04	5.00	2.70	2.52	3.92	4.65	2.41	1.83	2.98
**Inter-assay (%CV)**	6.32	4.19	5.08	5.73	9.71	5.76	9.12	10.89	8.01

Supplemental Table 3: Transformations of Alzheimer's disease-related outcome measures that were utilized in linear regression analysis

Alzheimer's disease-related outcome measure	Transformation
CAA score	Square root
apoE TBS	Square root
apoE TX	Square root
apoE FA	Natural logarithm
Aβ40 TBS	Natural logarithm
Aβ40 TX	Natural logarithm
Aβ40 FA	Natural logarithm
Aβ42 TBS	Natural logarithm
Aβ42 TX	Natural logarithm
Aβ42 FA	Natural logarithm
Aβ40 TBS/Aβ42 TBS ratio	Natural logarithm
Aβ40 TX/Aβ42 TX ratio	Natural logarithm
Aβ40 FA/Aβ42 FA ratio	Natural logarithm
Total tau TBS	Square root
Total tau TX	Square root
Total tau FA	Square root
p-tau TBS	Square root
p-tau TX	Natural logarithm
p-tau FA	Natural logarithm
CLDN5 TX/CD31 TX	None
OCLN TX/CD31 TX	Square root

Supplemental Table 4. Associations of sex and *APOE* ε4 with CAA score.

		Association with CAA score			
		Unadjusted analysis		Adjusting for age at death, sex[1], number of *APOE* ε4 alleles[2], Braak stage, and Thal phase	
Variable	N	Regression coefficient (95% CI)	P-value	Regression coefficient (95% CI)	P-value
Male sex					
All patients	469	0.09 (0.01, 0.16)	0.020	**0.11 (0.04, 0.19)**	**0.002**
Carriers of *APOE* ε4	311	0.10 (0.01, 0.18)	0.030	**0.12 (0.04, 0.21)**	**0.006**
Non-carriers of *APOE* ε4	158	0.10 (-0.04, 0.23)	0.15	0.11 (-0.03, 0.24)	0.13
Presence of the *APOE* ε4 allele					
All patients	469	**0.23 (0.16, 0.31)**	**<0.001**	**0.21 (0.14, 0.29)**	**<0.001**
Males	224	**0.24 (0.13, 0.34)**	**<0.001**	**0.19 (0.08, 0.30)**	**0.001**
Females	245	**0.24 (0.13, 0.35)**	**<0.001**	**0.23 (0.12, 0.34)**	**<0.001**

CI=confidence interval. Regression coefficients, 95% CIs, and p-values result from linear regression models, where CAA score was considered on the square root scale. Regression coefficients are interpreted as the change in mean CAA score (on the square root scale) corresponding to presence of male sex or to each additional *APOE* ε4 allele. [1] Sex was not adjusted for in analysis of only males or females. [2] Number of *APOE* ε4 alleles was not adjusted for in analysis of only carriers or non-carriers of *APOE* ε4. P-values <0.025 were considered as statistically significant after applying a Bonferroni correction for multiple testing given the two different variables that were assessed for association with CAA score; statistically significant associations are shown in bold.
